# Menopausal Transition: Prospective Study of Estrogen Status, Circulating MicroRNAs, and Biomarkers of Bone Metabolism

**DOI:** 10.3389/fendo.2022.864299

**Published:** 2022-05-13

**Authors:** Jiri Baloun, Aneta Pekacova, Laszlo Wenchich, Hana Hruskova, Ladislav Senolt, Xiao Svec, Karel Pavelka, Jan J. Stepan

**Affiliations:** ^1^ Institute of Rheumatology, Prague, Czechia; ^2^ Department of Rheumatology, First Faculty of Medicine, Charles University in Prague, Prague, Czechia; ^3^ Department of Obstetrics and Gynecology, First Faculty of Medicine, Charles University in Prague, Prague, Czechia; ^4^ General University Hospital in Prague, Prague, Czechia

**Keywords:** estrogen, circulating miRNA, bone remodeling, oophorectomy, osteoporosis prevention and control

## Abstract

**Objective:**

Osteoporosis is associated with an impaired balance between bone resorption and formation, which in turn leads to bone loss and fractures. Many recent studies have underlined the regulatory role of microRNAs (miRNAs) in bone remodeling processes and their potential as biomarkers of osteoporosis. The purpose of this study was to prospectively examine the association of circulating miRNAs and bone biomarkers with estrogen status in women before and after oophorectomy, as well as in oophorectomized women on estrogen therapy.

**Methods:**

In this prospective study, we included 11 women before oophorectomy and hysterectomy and at 201 ± 24 days after the surgery. Another 11 women were evaluated 508 ± 127 days after oophorectomy and hysterectomy and after an additional 203 ± 71 days of estradiol treatment. Serum miRNAs were profiled by sequencing. Estrogen status and biomarkers of bone metabolism were quantified. Bone mineral density was assessed in the lumbar spine.

**Results:**

Our analysis revealed 17 miRNAs associated with estrogen levels. Of those miRNAs that were upregulated with estrogen deficiency and downregulated after estrogen therapy, miR-422a correlated with serum beta-carboxy-terminal type I collagen crosslinks (β-CTX) and procollagen 1 N-terminal propeptide (P1NP); and miR-1278 correlated with serum β-CTX, P1NP, osteocalcin, sclerostin, and Dickkopf-1(Dkk1). In contrast, we found an inverse association of miR-24-1-5p with estrogen status and a negative correlation with serum β-CTX, P1NP, osteoprotegerin, and sclerostin levels.

**Conclusion:**

The reported miRNAs associated with estrogen status and bone metabolism could be potential biomarkers of bone pathophysiology and would facilitate studies on the prevention of postmenopausal osteoporosis. Our findings require validation in an extended cohort.

## Introduction

Osteoporosis is a skeletal disease characterized by low bone mass and microarchitectural deterioration, which are related to unbalanced bone resorption and bone formation (1), leading to bone fragility and susceptibility to fracture (2). The consequences of osteoporosis substantially increase the consumption of medical and economic resources worldwide (3). Until now, many expression profiling studies have revealed that bone remodeling processes are well-tuned at the transcriptional level and controlled by noncoding RNAs, especially long non-coding RNAs and microRNAs (miRNAs) (4–6).

MiRNAs are short RNAs of typically 18-22 nucleotides that operate as post-transcriptional regulators of protein-coding genes and the non-coding genome (7). They regulate many developmental and functional pathways, and their aberrant expression has been associated with various disorders (8), including osteoporosis (7, 9). In particular, several miRNAs with distinguishable expression profiles have been identified in bone samples from patients with osteoporosis and those with low impact fractures in most studies (10–15). Generally, during the bone remodeling process, miRNAs regulate differentiation of osteoblast and osteoclast and bone formation by targeting the regulators of osteogenesis or osteoclastogenesis, namely, transcription factors and signaling pathways (7, 16, 17). MiRNAs can escape from tissues into the bloodstream and become circulating miRNAs, providing additional information related to bone metabolism (7). The existing literature has mainly assessed the profiles of circulating miRNAs in patients with established osteoporosis and low-impact fractures. Recent research has drawn attention to the association of circulating miRNAs with bone remodeling in menstrual cessation (18). In the early postmenopausal period, the bone mineral density is not decreased but the bone resorption rate excessively exceeds new bone formation, posing a possible osteoporosis risk (19). The purpose of this study was to prospectively examine the association of circulating miRNAs and bone biomarkers with estrogen status in women before and after oophorectomy, as well as in oophorectomized women on estrogen therapy.

## Patients and Methods

### Patients

Between July 2018 and May 2019, 22 women who had been consecutively referred for bone status assessment through the Department of Obstetrics and Gynecology, General University Hospital in Prague, and had hysterectomy and bilateral oophorectomy before menopause were included in the present study. Surgery was indicated for benign gynecological diseases, including leiomyoma (14), metrorrhagia (3), breast cancer gene mutation (3), dysplasia of the cervix (1), and benign ovarian cyst (1). Of these subjects, 11 women were evaluated 18 ± 10 days before oophorectomy and hysterectomy and 201 ± 24 days after the surgery. None of these women received hormone therapy after surgery. The other 11 women who had previously undergone oophorectomy and hysterectomy were evaluated at baseline 508 ± 127 days after the surgery. Hormone treatment was initiated by their gynecologist. A check-up was performed after 203 ± 71 days of oral hormonal treatment, corresponding to 1 mg of estradiol per day.

Complete medical histories and dietary questionnaires were obtained from all subjects. No subject had bone- or calcium-related metabolic disease or received medications that are known to affect bone or calcium metabolism. No woman had a history of alcohol abuse, diabetes mellitus, active neoplastic disease, liver disease, known endocrine and rheumatologic disease, immunosuppressive treatment, treatment with corticosteroids, aromatase inhibitors, anti-osteoporotic drugs, anticonvulsants, or a history of fragility fractures or had previously received hormone replacement therapy. Subjects were advised to maintain their usual physical activity and dietary pattern throughout the study.

The study was approved by the Institutional Review Board at the Institute of Rheumatology, Prague, Czech Republic. All participants signed written informed consent forms to participate and agreed to DXA and blood tests. All study procedures were carried out in compliance with the laws and regulations governing the use of human subjects (Declaration of Helsinki) (20).

### Blood Collection

Venous blood samples were obtained after an overnight fast from each subject at baseline and follow-up visits for laboratory analyses. To separate the serum, whole blood was collected into commercially available collection tubes. After one hour of clotting, the blood was centrifuged at 2000xg for 20 minutes. Serum (supernatant) was transferred into a new tube and frozen at -70°C until further processing.

### MiRNA Isolation

MiRNAs were extracted from 200 µL of blood serum using NucleoSpin miRNA Plasma (Macherey-Nagel) according to the manufacturer’s protocol. Isolated miRNAs were not quantified since concentrations were below the detection limits of the NanoDrop 2000. Follow-up procedures were conducted with undiluted samples.

### Massively Parallel Sequencing

#### Library Preparation

Libraries for massively parallel sequencing (MPS) were prepared from 10.5 µL of isolated miRNAs using Kit v3 (Bioo Scientific) according to the manufacturer’s protocol. Fragments after the amplification step were analyzed using a Fragment Analyzer (Advanced Analytical), and fragments of the miRNA library (145 bp) were quantified. Samples were pooled in equal concentrations of miRNA library fragments, which were isolated using the Pippin Prep system with 3% agarose (Sage Science) before sequencing. The isolated fragments were quantified using a Qubit 2.0 fluorometer (Thermo Fisher Scientific) and were used for sequencing on a NextSeq 500 (Illumina) according to the manufacturer’s protocol.

### Bioinformatics Analyses

Adaptor sequences in MPS data were identified and removed with Cutadapt (v2.5) software (21). Only high-quality reads with a length between 16 and 28 bp after adapter trimming were retained as potential miRNA reads. The quality of both raw and processed reads was evaluated using FastQC software (22).

Count-based miRNA expression data were generated by the Chimira tool (23) from FASTQ files. All sequences were adapter trimmed and mapped against miRBase v22 (24), allowing up to two mismatches per sequence. All samples were evaluated for differential expression using DESeq2 (25). Further analyses were performed using R/Bioconductor packages. Raw data and annotated sequences of the small RNA libraries were uploaded to the GEO database.

DESeq2 computed the normalized miRNA-read count, dispersion, and base mean (the average of the normalized count values, divided by the size factors, taken over all samples). The dispersion can be understood as a squared coefficient of variation and the mean dispersion value of 26.04 represents the coefficient of variation of 4.49. miRNAs with a base mean < 10 were indistinguishable from the sampling noise and were filtered out of the dataset.

### Bone Densitometry

The areal bone mineral density (aBMD) of the lumbar spine (LS-BMD) was evaluated using dual-energy X-ray absorptiometry (DXA) (GE Healthcare Lunar software version 14.1) and expressed in grams/cm^2^ and T-score. The T-score was calculated using the National Health and Nutrition Examination Survey (NHANES) of young women as a reference. Quality control assurance measurements were performed following the manufacturer’s recommendations. The short-term *in vivo* precision error for the lumbar spine (L1–L4) was 0.7%; the long-term *in vivo* precision error was 0.31%. Trained examiners with extensive experience conducted the measurements.

### Biochemical Analysis

The concentrations of total serum calcium, phosphate, glucose, total alkaline phosphatase, γ-glutamyltransferase (GGT), thyroid-stimulating hormone (TSH), 1-84 amino acid fragment of parathyroid hormone (intact PTH), 25(OH)D, beta-carboxy-terminal type I collagen crosslinks (β-CTX), procollagen 1 N-terminal propeptide (P1NP), osteocalcin, estradiol, and FSH were determined using the Beckman Coulter AU 680 (Beckman Coulter, USA), Roche cobas e601 (Roche, Switzerland), and Liaison XL (Diasorin, Italy) analytical systems. The plasma estradiol measuring range was 18.4-11,000 pmol/l with intra- and interassay CV < 12%. The plasma FSH measuring range was 0.1-200 IU/l with intra- and interassay CV < 6%. The serum β-CTX measuring range was 0.01-6 µg/l with intra- and interassay CV < 6%. The serum PINP measuring range was 5-1200 µg/l with intra- and interassay CV < 6%. The estimated glomerular filtration rate (eGFR) was calculated (26). Serum sclerostin was assessed using the Bioactive Sclerostin ELISA (Biomedica, Austria), and the measuring range was 10-320 pmol/l with intra- and interassay CV < 5%. Serum Dickkopf-1 (DKK1) was analyzed using the DKK-1 ELISA (Biomedica, Austria), and the measuring range was 10-160 pmol/l with intra- and interassay CV < 3%. Serum osteoprotegerin was measured using the Human Osteoprotegerin ELISA (Biovendor, Czech Republic), and the measuring range was 1.5-60 pmol/l with intra- and interassay CV < 7%.

### Biostatistical Analyses

Characteristics of the population were computed using SigmaPlot version 14.0, Systat Software, San Jose, CA, USA. The Kolmogorov–Smirnov test was applied to assess the normality of the data. Data with a normal distribution are presented as the mean ± SD, while nonparametric data are presented as the median and quartiles. The paired t-test was used to compare biochemical variables, depending on the normality of the variables. Univariate analysis with Pearson correlation was used. Statistical significance was defined as a p-value < 0.05.

DESeq2 algorithm provided a matrix of normalized read counts, which were analyzed with RStudio software and relevant packages (27, 28). Since miRNAs had normalized read count equal to zero in several samples, we employed glmmTMB R package (28), in which we can specify fixed and random effects models for the conditional and zero-inflated components of the model with negative binomial assumption. Using this R extension, we fitted a GLMM-NB, using the normalized read count of miRNAs as the outcome variable, with fixed effects of the level of estrogen (or the biomarker of bone metabolism) and follicle-stimulating hormone (FSH) as a confounding variable. We also fitted a random-effects structure, which includes a random intercept for patient ID. To test differential miRNA expression between patients with optimal and low estrogen levels (estrogen status) or the association with the biomarkers of bone remodelling (adjusted with FSH level and a random intercept for patient ID), the following model was used:


$$\text{log}({µ}_{ij})=(\beta_{0}+\;\gamma_{0j})+\;\beta_{1}(estrogen\;status\;or\;biomarker)+\;\beta_{2}(FSH)+\;\epsilon_{ij}$$

$$normalized\;read\;counts\;\sim \;NB(u_{ij},\theta )$$

where *i* - the miRNA; *j* – patients ID; *β* – regression coefficient; *γ* – random parameter; and *ϵ* – error. The effect of estrogen level was represented as the incidence rate ratio (IRR), which means that the number of expected observations on the miRNAs’ count changes by the value of IRR if the estrogen level changes from optimal to low. The association of the bone modelling biomarkers is represented as the slope, which is the change of normalized read counts if the value of the biomarkers changes by one unit. The adjusted p-value was calculated using Benjamini-Hochberg (BH) method (29) and indicated in **Supplementary Tables**.

## Results

### Demographic and Clinical Characteristics

**Figure 1:**
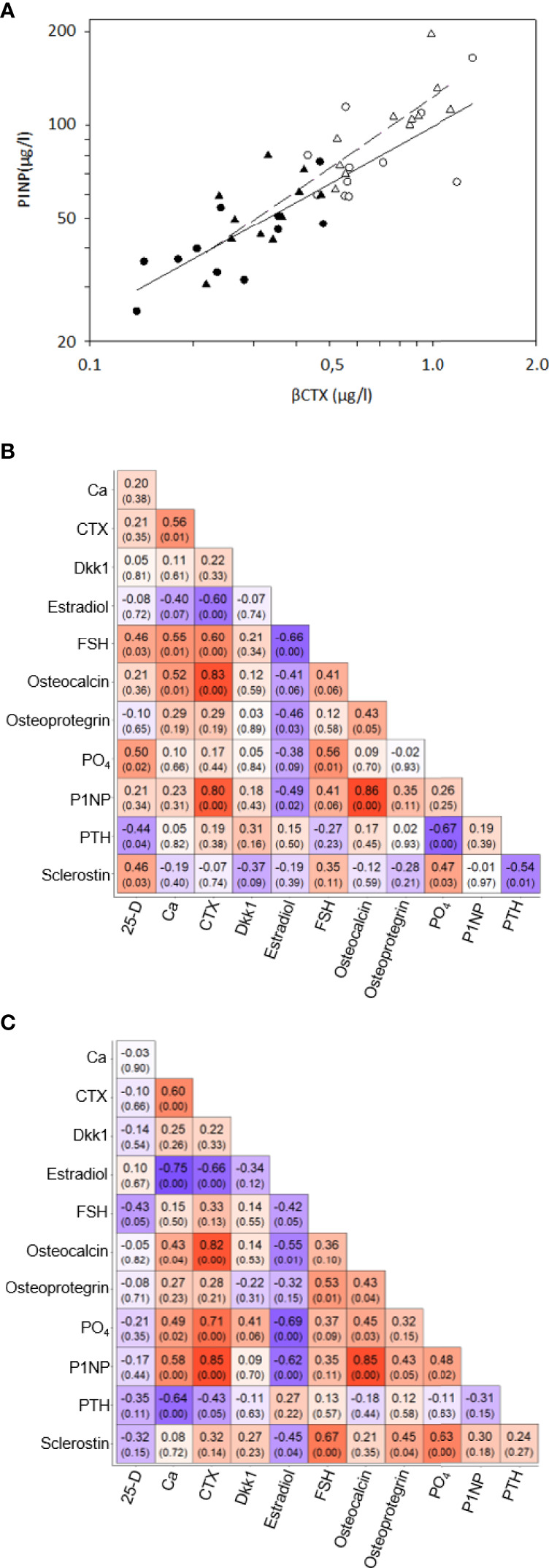
Correlations of bone remodeling biomarkers – **(A)** Correlations between serum βCTX and P1NP in 11 women before oophorectomy, Group A (•) and untreated after surgery, Group B (o) (solid line), and in 11 oophorectomized women untreated, Group C (Δ) and treated with estradiol, Group D (▲). **(B)** Correlation matrix of selected the bone remodelling biomarkers in the Group A and B. The matrix included Pearson’s correlation coefficients and p-value in the bracket below. The color indicates the strength and direction of the coefficient. **(C)** Correlation matrix of selected the bone remodelling biomarkers in the Group C and D. The matrix included Pearson’s correlation coefficients and p-value in the bracket below. The color indicates the strength and direction of the coefficient.

At baseline, no differences were observed in age, body mass index (BMI), serum 1-84 amino acid fragment of parathyroid hormone (intact PTH), creatinine, glucose, thyroid stimulating hormone (TSH), or creatinine clearance between women before and after oophorectomy (Groups A and C, **Table 1**). At follow-up 201 days after oophorectomy (Group B), lumbar spine bone mineral density (LS BMD) and serum estradiol decreased significantly, while serum follicle-stimulating hormone (FSH), type 1 collagen crosslinked C-telopeptide (β-CTX), intact amino-terminal propeptide of type I procollagen (P1NP), alkaline phosphatase, osteocalcin, sclerostin, calcium, and phosphate significantly increased compared to values before oophorectomy (Group A). Similar differences were observed between the variables before oophorectomy (Group A) and 508 days after oophorectomy (Group C), except for sclerostin. In women on oral hormonal therapy for 203 days (from 508 days, Group C, to 711 days after oophorectomy, Group D), LS-BMD and serum estradiol increased significantly, while serum FSH, β-CTX, P1NP, alkaline phosphatase, osteocalcin, sclerostin, osteoprotegerin, calcium, phosphate, and PTH decreased. Dkk1and OPG serum levels did not correlate with bone remodeling markers (β-CTX and P1NP), while levels of β-CTX and P1NP were highly correlated (**Table 2**; **Figure 1**).

**Table 1:** Characteristics of 11 untreated women prior to oophorectomy (A) and 201 days after oophorectomy (B) and 11 untreated women 508 days after oophorectomy (C) and then after 203 days of estradiol treatment (D).

Characteristics	A	B	p	C	D	p
	Prior to oophorectomy	After oophorectomy		Untreated after oophorectomy	Treated after oophorectomy	
Age (years)	47.9 (2.5)	48.5 (2.4)	<0.001	48.6 (5.1)	49.3 (5.2)	<0.001
BMI (kg/m^2^)	29.7 (26.3 - 34.5)	29.2 (25.5 - 35.3)	0.766	24.3 (20.8 - 31.1)	23.6 (21.0 - 31.2)	0.388
Spine BMD (g/cm^2^)	1.327 (0.140)	1.266 (0.139)	<0.001	1.012 (0.181)	1.048 (0.183)	<0.001
βCTX (µg/l)	0.24 (0.18 - 0.36)	0.57 (0.55 - 0.92)	<0.001	0.86 (0.54 - 0.99)	0.33 (0.26 - 0.41)	<0.001
PINP (µg/l)	39.8 (33.4 - 51.0)	73.2 (59.7 - 109.8)	<0.001	104.5 (74.3 - 111.9)	50.7 (42.7 - 60.9)	<0.001
Osteokalcin (µg/l) Osteoprotegerin (pmol/l)	17.0 (13.3 - 19.2)	22.4 (20.1 - 27.7)	<0.001	32.4 (25.3 - 38.2)	22.2 (19.6 - 27.6)	0.002
Osteoprotegerin (pmol/l) Sclerostin (pmol/l)	5.19 (3.61)	6.34 (1.62)	0.067	5.57 (1.40)	4.79 (1.36)	0.002
Sclerostin (pmol/l) DKK1	111.9 (66.0 - 157.2)	133.1 (112.7-177.5)	0.001	120.3 (111.6 - 134.9)	95.1 (78.5 - 106.9)	0.001
DKK1 (pmol/l)	46.2 (17.8)	56.0 (19.2)	0.012	54.6 (13.5)	46.0 (14.4)	0.014
Phosphate (mmol/l) Calcium	1.06 (0.12)	1.22 (0.14)	0.004	1.31 (0.09)	1.07 (0.11)	<0.001
Calcium (mmol/l)	2.42 (0.10)	2.52 (0.16)	0.042	2.47 (0.05)	2.36 (0.09)	0.001
Intact PTH (pmol/l) 25(OH)D	3.12 (1.20)	2.92 (1.54)	0.470	2.38 (0.85)	2.89 (0.86)	0.037
25(OH)D (nmol/l)	46.8 (22.8)	63.2 (17.3)	0.045	71.3 (20.1)	78.5 (14.6)	0.346
Estradiol (pmol/l)	338.5 (188.3)	36.3 (18.2)	<0.001	22.3 (5.8)	161.4 (66.3)	<0.001
FSH (IU/l)	6.6 (4.2)	86.1 (31.6)	<0.001	111.0 (51.8)	65.5 (38.5)	<0.001
Glucose (mmol/l)	5.2 (0.5)	5.3 (0.5)	0.739	5.3 (0.4)	5.2 (0.5)	0.098
ALP (µkat/l)	1.22 (0.99 - 1.57)	1.69 (1.54 - 2.8)	<0.001	1.45 (1.20 - 1.78)	1.05 (1.01 - 1.34)	<0.001
TSH (mIU/l)	2.32 (2.00 - 3.73)	3.13 (1.41 - 4.36)	0.803	2.36 (1.78 - 4.93)	2.18 (1.83 - 3.27)	0.767
eGFR (ml/sec/1.73 m^2^)	1.60 (0.12)	1.66 (0.22)	0.139	1.57 (0.17)	1.60 (0.20)	0.535

Data are the mean (SD) or median (IQR).

**Table 2:** Association of changes in 17 miRNAs with disparate concentrations between sufficient and low estrogen levels in serum with biomarkers in 11 untreated women before oophorectomy and 201 days after, and in 11 women initially untreated for 508 days and then treated with estradiol for 203 days.

miRNA	Estradiol	βCTX	PINP	Sclerostin	Dkk1	Osteoprotegrin
miR-195-5p	2.12	0.22	0.00	0.00	0.00	-0.06
	**0.048**	0.759	0.370	0.502	0.832	0.381
miR-196a-5p	162.96	2.54	0.01	0.02	0.07	0.25
	**0.008**	0.390	0.549	0.482	**0.001**	0.605
miR-200a-3p	4.92	1.78	0.01	0.00	0.03	-0.01
	**0.028**	0.350	0.665	0.852	0.114	0.929
miR-424-3p	3.41	1.68	0.01	0.00	-0.01	0.00
	**0.050**	0.138	0.133	0.596	0.746	0.972
miR-505-5p	1262.52	7.67	0.02	-0.06	-0.01	0.11
	**0.033**	0.095	0.402	0.167	0.889	0.756
miR-550a-3-5p	225659839.00	-6.19	-0.03	0.02	-0.19	-2.75
	**0.002**	0.674	0.725	0.782	0.352	0.226
miR-3120-3p	12.03	1.88	0.02	0.00	0.02	0.24
	**0.032**	0.427	0.191	0.874	0.679	0.441
miR-100-5p	3.19	1.30	0.01	0.00	0.02	-0.04
	**0.018**	0.183	0.094	0.724	0.112	0.733
miR-122-5p	5.23	1.57	0.02	0.00	0.04	-0.12
	**0.045**	0.383	0.136	0.998	0.162	0.616
miR-1278	413.22	11.70	0.04	0.06	0.18	-0.45
	**<0.001**	**0.002**	**<0.001**	**0.035**	**0.002**	0.176
miR-1304-5p	25.08	5.05	0.04	-0.01	-0.02	-0.34
	**0.004**	0.089	0.168	0.688	0.831	0.147
miR-422a	2.21	1.29	0.01	0.00	-0.01	0.05
	**0.013**	**0.015**	**0.001**	0.669	0.501	0.475
miR-566	42.85	3.20	0.01	0.02	0.00	-0.07
	**0.018**	0.246	0.483	0.573	0.940	0.873
let-7d-3p	1.89	1.03	0.01	0.00	0.01	-0.07
	**0.038**	0.053	0.259	0.904	0.307	0.271
miR-132-5p	0.16	-1.91	-0.01	0.00	0.00	-0.15
	**0.034**	0.079	0.322	0.713	0.951	0.177
miR-24-1-5p	0.00	-6.71	-0.05	-0.04	-0.02	-1.11
	**<0.001**	**0.006**	**0.020**	**0.029**	0.766	**<0.001**
miR-619-5p	0.07	-2.60	-0.01	-0.01	-0.09	-0.10
	**0.011**	0.088	0.141	0.621	**0.007**	0.511

Data are presented as the incident rate ratios and p values for estradiol (bold). Data for biomarkers are presented as the slope (β) and p-values.

Of the 48 sequenced samples, 22 samples were paired up as having sufficient and low levels of estrogen. The four remaining samples were additional measurements to four paired samples and were thus discarded.

### MiRNome Profiling in Patient Serum – Sequencing Study

Sequencing revealed a median coverage of over 10 million unprocessed reads per sample, but after trimming and aligning to miRBase v22, we found that the median was 4.9 million reads per sample with a balanced distribution (SD = 0.043).

After alignment, we found 1305 unique miRNA sequences. Since low-read count miRNAs with high dispersion might be indistinguishable from sampling noise and increase the false-positive rate, we filtered out all miRNAs with a base mean of > 10 and found 439 miRNAs.

Since patients were paired up, we employed generalized linear mixed-effects modeling with the negative binomial assumption (GLMM-NB) and patients as the random effect. The first analysis focused on changes in miRNA concentrations in serum with sufficient and low levels of estrogen, and we found 17 miRNAs with *p* < 0.05 and a difference in fold change of > 50%. To affirm their connection to osteoporosis, we examined their relationship to lumbar spine BMD, biomarkers and hormones associated with bone metabolism, and biomarkers of low-grade inflammation. Significant associations are given in **Table 2** and **Tables S1**, **S2** in detail.

**Figure 2:**
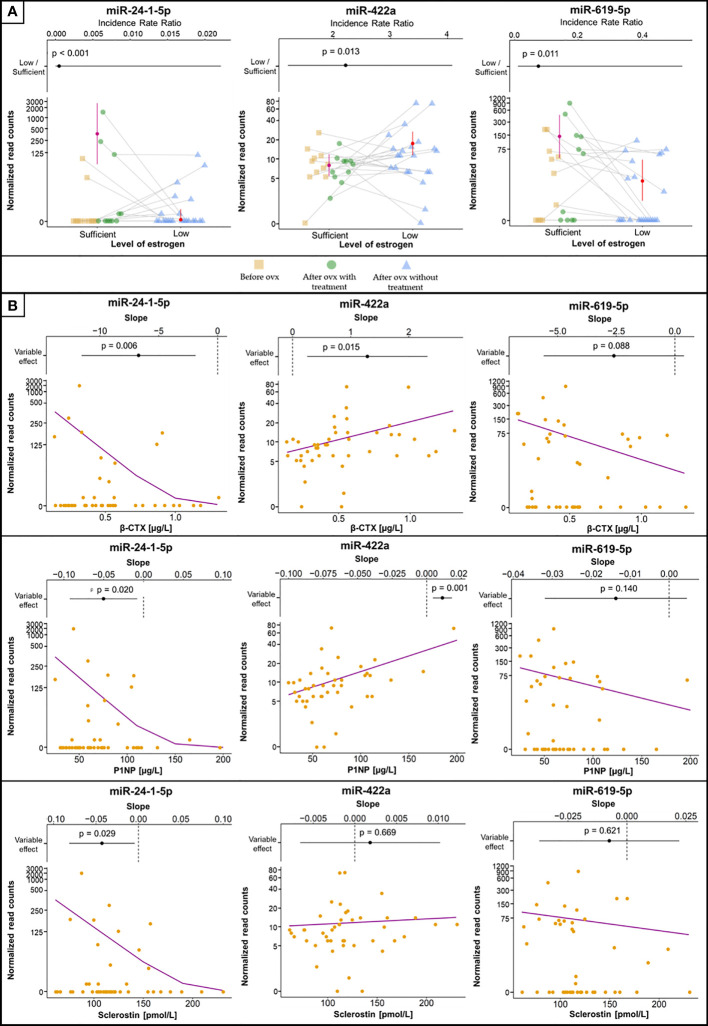
Selected miRNAs associated with estrogen status and biomarkers of bone metabolism or inflammation. **(A)** MiRNA concentrations between groups, with error bars representing 95% confidence intervals and estimated means (filled circles). Samples from the same patient are connected with grey lines. The plots at the top are visualizations of the 95% confidence intervals with IRR (filled circles), where values > 1 indicate increased concentrations in samples with low estrogen levels and vice versa. The vertical dashed line indicates IRR equal to 1 (= no difference). p values denoting the statistical significance between the groups are specified above the slope and were computed using GLMM-NB. **(B)** The association of selected miRNAs with levels of biomarkers. The violet line is the regression line computed using GLMM-NB. The plots at the top are visualizations of 95% confidence intervals with the slope (β) (filled circles), where a positive value indicates a positive association of the miRNA with the biomarker and vice versa. The vertical dashed line indicates a slope equal to 0 (= no difference). p values denoting the statistical significance of the association are specified above the slope and were computed using GLMM-NB.

The concentrations of 14 miRNAs showed an inverse association with changes in estrogen status (**Table 2**). Serum concentrations of miR-422a were positively associated with serum β-CTX and P1NP levels (**Figure 2**). The amount of miR-1278 was positively associated with serum β-CTX, P1NP, sclerostin, osteocalcin, and Dkk1 levels. Increased miR-422a levels were associated with increased serum β-CTX and P1NP levels (**Table 2**).

In contrast, the levels of miR-132-5p, miR-24-1-5p, and miR-619-5p showed a positive association with estrogen status (**Figure 2**). Of these, miR-24-1-5p was negatively associated with β-CTX, PINP, osteoprotegerin, and sclerostin levels. The remaining miRNAs were not associated with any biomarker of bone metabolism or inflammation. Furthermore, the associations between miRNA concentrations and lumbar spine BMD were not significant.

### MiRNome Profiling in Patient Serum – osteomiR^®^

An association between osteoporosis and circulating miRNA profiles can be assessed using commercially available kits, such as osteomiR^®^ (TAmiRNA, Austria). This complete kit includes materials for miRNA isolation, reverse transcription, and qRT–PCR with LNA probes. In addition to quality control (QC) probes, this kit screened ten miRNAs connected to osteoporosis. However, in most of our samples, these miRNAs were below the detection limit of qRT–PCR (C_T_ > 30), and the QC probes indicated low concentrations after isolation. We verified these results by additional miRNA isolation from serum and quantification by TaqMan Advanced miRNA assays (Thermo).

All ten miRNAs included in the osteomiR^®^ kit were detected in our miRNA-Seq analysis, and their relation to estrogen levels and biomarkers was assessed using GLMM-NB with patients as a random effect and FSH as a confounding variable. Nonetheless, no miRNA was significantly different between samples with sufficient and low estrogen levels (**Table S3**), but we found an association of miR-375 with β-CTX and P1NP (**Table S4**).

## Discussion

In this study, we screened the profile of circulating miRNAs in women before and after oophorectomy, as well as in oophorectomized women on estrogen therapy. We identified 17 miRNAs, which were different in sera with either sufficient or low estrogen levels. Of the 14 miRNAs upregulated after oophorectomy and downregulated with estrogen therapy, miR-1278, miR-24-1-5p, and miR-422awere associated with biomarkers of bone metabolism, suggesting their role in the pathogenesis of osteoporosis (11, 30–33).

The expression of miR-422a was previously detected in monocytes and was considered a potential biomarker for postmenopausal osteoporosis (34). We observed a decrease in miR-422a levels after estradiol treatment, and a positive correlation of miR-422a levels with β-CTX and PINP but not with sclerostin levels. These findings support the role of miR-422a expression in bone remodeling associated with estrogen status (35–39).

The linkage of miR-1278 to bone metabolism was not explored previously, but we found associations of miR-1278 with serum levels of bone remodeling biomarkers (β-CTX, P1NP, and osteocalcin). In addition, miR-1278 was positively associated with sclerostin and Dkk1, which are inhibitors of the WNT signaling pathway. These associations are in concordance with previous studies on the association between inhibitors of the WNT signaling pathway and estrogen status (40–44). The Wnt/β-catenin pathway activation enhances bone mass not only by stimulating osteoblastogenesis but, at least to some extent, also by inhibiting osteoclastogenesis as well (44).

In contrast, estrogen deficiency in our patients was associated with decreased serum levels of miR-24-1-5p and increased serum levels of β-CTX, P1NP, and sclerostin compared to those of women with sufficient estrogen status. Similarly, postmenopausal women treated with teriparatide had a significant and inverse correlation of miR-24-3p at 3 months with P1NP at 3 and 12 months and with β-CTX at 12 months (45). However, Seeliger et al. observed an upregulated miR-24-3p concentration in sera and bone tissue of osteoporotic patients (11), while another study did not find any differences in the relative expression of miR-24-3p between healthy, osteoporotic, and sarcopenic postmenopausal women (14). The discrepancy observed among these studies might arise from the quantification of different isoforms of this miRNA.

In this study, levels of β-CTX and P1NP were highly correlated, reflecting the coupling of bone resorption and bone formation (46). The early phase after oophorectomy is characterized by the prevalence of bone resorption over bone formation; following initiation of estrogen treatment, a decrease in markers of bone resorption is later followed by a decrease in markers of bone formation (47). Compared with women before oophorectomy and women treated with estrogen, untreated oophorectomized women showed higher serum β-CTX, PINP, and osteocalcin, but also higher sclerostin, Dkk1, and OPG, and levels. Our sclerostin data correspond with a negative correlation of sclerostin levels with the free estradiol index in postmenopausal women (48), with significantly higher serum sclerostin levels in postmenopausal women than premenopausal women (49), and with a decrease in serum (42, 50) and bone mRNA sclerostin levels (43) after the administration of estrogen. DKK1 expression in bone was enhanced after ovariectomy in mice, and DKK1 antisense oligonucleotide treatment reduced the promoting effect of estrogen deficiency on DKK1 (51). Postmenopausal women with significantly increased serum DKK1 had more significant osteoporosis (51). Osteoprotegerin levels are higher in postmenopausal osteoporotic women, compared with controls (52), and the inverse relationship between serum OPG and serum oestradiol levels was demonstrated in females (53). Serum OPG levels measured after 3 months and 1 year of HT decreased significantly compared to baseline (54).

In this study, while miR-1278, miR-24-1-5p, and miR-422a correlated with markers of bone remodeling (β-CTX and P1NP), only miR-1278 and miR-24-1-5p correlated with serum sclerostin levels. Both in our untreated and treated women, serum levels of inhibitors of bone formation (sclerostin and Dkk1), and bone resorption (OPG), were not correlated with markers of bone remodeling. Interestingly, circulating sclerostin levels do not decrease in postmenopausal women on antiresorptive therapy with bisphosphonates (55). Taken together, in agreement with previous data (49, 54), our results indicate that changes in serum sclerostin, Dkk1 and OPG levels may represent a compensatory response of the osteocyte functional activity reflecting acute estrogen deficiency and/or estrogen replete state, rather than changes in remodeling of bone.

Above all, the amount of miR-200a-3p was elevated in our patients with low estrogen levels and downregulated in women with high estrogen. Physiologically, miR-200s are overexpressed in several clinical conditions related to estrogen status, such as in the mammary glands during mammary gland development, pregnancy, and lactation (56), as well as in estrogen-dependent cancers (57). In humans, miR-200a-3p was associated with osteoporosis (4), albeit not significantly expressed in patients with osteoporotic bone fractures (11, 13), whereas our GLMM-NB analysis did not reveal any pertinence of miR-200a-3p to lumbar spine BMD or biomarkers. This particular miRNA is notable due to its substantial increase in our patients with low estrogen levels and its apparent relevance to osteoporosis.

Our study did not aim to establish an association of miRNAs with the probability of osteoporosis and low impact fractures. To assess osteoporosis in our cohort, we used the commercially available kit osteomiR^®^ (TamiRNA) (58). However, the screening failed due to an insufficient amount of miRNAs after isolation, and we speculate that our serum samples contained insufficient levels of miRNA for qRT–PCR. Fortunately, our well-designed MPS could detect miRNAs at low concentrations, so we were able to quantify all miRNAs included in osteomiR^®^. This underlines the importance of sample processing and highlights the advantage of MPS.

Although our study is informative, there are several limitations. First, several lifestyle factors that can influence estrogen levels, such as nutritional status and exercise, were not addressed. Second, the miRNAs of interest had zero counts in several samples; however, we employed a statistical approach, which should resolve this problem. Third, the cohort of 22 pairs of samples might be insufficient for a proper statistical analysis, but recruitment of such a uniform cohort is complicated and time-consuming. Fourth, the duration of our study was relatively short, as it was an exploratory study. Given the short duration of the study and the low number of cases, changes in BMD should be interpreted with caution. Fifth, the ovariectomized women in this study were treated with 1 mg of estradiol. Bone remodeling is dose-dependently regulated by estrogen (59, 60). Estradiol at follicular to periovulatory levels is needed to suppress a mildly activated immune system responsible for increased postmenopausal bone resorption (59). However, women in this study were referred for bone status assessment, and the estrogen therapy was prescribed by their gynecologists. Sixth, the MPS results were not validated by qRT–PCR due to the low miRNA concentration in sera. Seventh, as smoking was reported by only one woman treated with estrogen after oophorectomy, the effects of smoking on the liver metabolism of sex hormones were not taken into consideration in the analyses. Further significant limitations include seasonal variability, vitamin D status, and the effects of PTH on osteocytic sclerostin production (61, 62). In our patients treated with estrogen, no significant correlation was observed between serum sclerostin, serum vitamin D and PTH. Six out of 11 women before oophorectomy were vitamin D insufficient. They were supplemented with 800 IU vitamin D. Previously, such a supplementation did not significantly change sclerostin levels in women (63). In patients with vitamin D severe deficiency (25- hydroxyvitamin D level ≤ 20 ng/mL) receiving a monthly intramuscular injection of 300,000 IU of cholecalciferol, serum sclerostin levels decreased (64). In our untreated patients, after oophorectomy, an increase in serum 25- hydroxyvitamin D was correlated with the increase in sclerostin levels. Finally, this study is descriptive, and our results warrant further validation by additional research.

To our knowledge, this is the first prospective study to compare the associations between changes in estrogen status and relative serum levels of circulating miRNAs with BMD and biomarkers of both bone metabolism in women before and after ovariectomy as well as in ovariectomized women after estrogen treatment. The design of this study eliminates the confounding effects of age on the association between the levels of circulating miRNAs, estradiol, follicle-stimulating hormone, and bone variables (65, 66). The present findings corroborate a few previous studies on the expression of miRNAs during treatment with anti-osteoporotic agents (45), as well as studies comparing profiles in postmenopausal osteoporotic and healthy premenopausal women (66, 67). Although this study reported substantial changes in several miRNAs (let-7d-3p, miR-1278, miR-24-1-5p, miR-422a, and miR-619-5p) that regulate osteoblast and osteoclast differentiation in association with estrogen status, these results require further research and validation in an extended cohort.

In summary, of the 14 miRNAs that showed upregulation with estrogen deficiency and downregulation after estrogen therapy, miR-422a correlated with serum β-CTX and P1NP and miR-1278 correlated with serum β-CTX, P1NP, osteocalcin, sclerostin, and Dkk1. Of the 3 miRNAs showing downregulation with estrogen deficiency and upregulation after estrogen therapy, miR-24-1-5p showed a negative correlation with serum β-CTX, P1NP, osteoprotegerin, and sclerostin levels. These miRNAs represent promising biomarkers in bone pathophysiology in studies on the prevention of postmenopausal osteoporosis and the mechanism of antiresorptive therapies. Future prospective studies are required to validate the potential clinical application of our findings.

## Data Availability Statement

The original contributions presented in the study are publicly available. This data can be found in GEO database (accession number GSE194086).

## Ethics Statement 

The studies involving human participants were reviewed and approved by The Institutional Review Board of the Institute of Rheumatology, Prague, Czechia. The patients/participants provided their written informed consent to participate in this study.

## Author Contributions

All authors were involved in drafting the article or in revising it critically for important intellectual content. All authors take responsibility for the integrity of the data and the accuracy of the data analysis JB was responsible for serum miRNA profiling, biostatistical analyses, data interpretation and manuscript preparation. AP was responsible for miRNA isolation and preparation of MPS libraries. LW was responsible for biochemical analyses and data interpretation. HH performed the clinical diagnoses. KP and LŠ performed the critical revision of the manuscript. XS was responsible for data interpretation. JS designed the study, was responsible for blood collection, patient follow-up, bone densitometry, immunoassays and manuscript preparation. All authors had access to the data, and have read and accepted the final version of the manuscript for submission.

## Funding

This research was funded by the Ministry of Health of the Czech Republic under grant NV18-05-00394 and by RVO 00023728 (Institute of Rheumatology), Charles University project SVV 260 523.

## Conflict of Interest

The authors declare that the research was conducted in the absence of any commercial or financial relationships that could be construed as a potential conflict of interest.

## Publisher’s Note

All claims expressed in this article are solely those of the authors and do not necessarily represent those of their affiliated organizations, or those of the publisher, the editors and the reviewers. Any product that may be evaluated in this article, or claim that may be made by its manufacturer, is not guaranteed or endorsed by the publisher.
