# Supplementary material for: Menopausal Transition: Prospective Study of Estrogen Status, Circulating MicroRNAs, and Biomarkers of Bone Metabolism
Source: Front Endocrinol (Lausanne). 2022 May 13;13:864299. doi: 10.3389/fendo.2022.864299 (PMC9137039; doi:10.3389/fendo.2022.864299)
Supplement: Supplementary file 1 [file Table_1.docx]

**Supplemental Table S1.** Associations between selected sequenced miRNAs and estrogen levels. The table includes only miRNAs with *p* < 0.05 and a difference in fold change > 50%. Data are described as IRR with 95% CI, t ratios, and *p* values. The difference in miRNA concentrations between samples with low/sufficient estrogen levels is presented as an IRR, where values > 1 indicate an increased concentration in samples with low estrogen levels and vice versa. The statistical model was adjusted for FSH. miRNAs are sorted in ascending order based on *p* values.

| **miRNA** | **Contrast** | **IRR** | **2.5% CI** | **97.5% CI** | **t-ratio** | **p-value** | **BH-adjusted p-value** |
| --- | --- | --- | --- | --- | --- | --- | --- |
| **miR-1278** | Low / Effective | 413,22 | 22,79 | 7492 | 4,205 | 0 | 0,062 |
| **miR-24-1-5p** | Low / Effective | 0 | 0 | 0 | -4,051 | 0 | 0,062 |
| **miR-550a-3-5p** | Low / Effective | 225659838,9 | 2366,42 | 2,15187E+13 | 3,393 | 0,002 | 0,279 |
| **miR-26a-5p** | Low / Effective | 0,94 | 0,9 | 1 | -3,249 | 0,002 | 0,313 |
| **miR-150-5p** | Low / Effective | 0,92 | 0,87 | 1 | -3,153 | 0,003 | 0,326 |
| **miR-1304-5p** | Low / Effective | 25,08 | 2,92 | 215 | 3,032 | 0,004 | 0,376 |
| **miR-4500** | Low / Effective | 1,35 | 1,09 | 2 | 2,87 | 0,007 | 0,495 |
| **miR-196a-5p** | Low / Effective | 162,96 | 4,17 | 6363 | 2,811 | 0,008 | 0,504 |
| **miR-619-5p** | Low / Effective | 0,07 | 0,01 | 1 | -2,681 | 0,011 | 0,625 |
| **miR-422a** | Low / Effective | 2,21 | 1,2 | 4 | 2,613 | 0,013 | 0,665 |
| **miR-29b-3p** | Low / Effective | 0,79 | 0,65 | 1 | -2,484 | 0,017 | 0,739 |
| **miR-100-5p** | Low / Effective | 3,19 | 1,24 | 8 | 2,473 | 0,018 | 0,739 |
| **miR-566** | Low / Effective | 42,85 | 1,96 | 938 | 2,463 | 0,018 | 0,739 |
| **miR-200a-3p** | Low / Effective | 4,92 | 1,19 | 20 | 2,277 | 0,028 | 0,886 |
| **let-7c-5p** | Low / Effective | 1,49 | 1,04 | 2 | 2,234 | 0,031 | 0,886 |
| **miR-3120-3p** | Low / Effective | 12,03 | 1,25 | 115 | 2,225 | 0,032 | 0,886 |
| **miR-505-5p** | Low / Effective | 1262,52 | 1,83 | 873369 | 2,209 | 0,033 | 0,886 |
| **miR-199b-3p** | Low / Effective | 1,19 | 1,01 | 1 | 2,196 | 0,034 | 0,886 |
| **miR-132-5p** | Low / Effective | 0,16 | 0,03 | 1 | -2,193 | 0,034 | 0,886 |
| **miR-101-3p** | Low / Effective | 1,06 | 1 | 1 | 2,184 | 0,035 | 0,886 |
| **let-7f-5p** | Low / Effective | 1,06 | 1 | 1 | 2,158 | 0,037 | 0,886 |
| **let-7d-3p** | Low / Effective | 1,89 | 1,04 | 3 | 2,151 | 0,038 | 0,886 |
| **miR-122-5p** | Low / Effective | 5,23 | 1,04 | 26 | 2,074 | 0,045 | 0,886 |
| **miR-195-5p** | Low / Effective | 2,12 | 1,01 | 4 | 2,046 | 0,048 | 0,886 |
| **miR-424-3p** | Low / Effective | 3,41 | 1 | 12 | 2,026 | 0,05 | 0,886 |
