# Supplementary material for: Menopausal Transition: Prospective Study of Estrogen Status, Circulating MicroRNAs, and Biomarkers of Bone Metabolism
Source: Front Endocrinol (Lausanne). 2022 May 13;13:864299. doi: 10.3389/fendo.2022.864299 (PMC9137039; doi:10.3389/fendo.2022.864299)
Supplement: Supplementary file 2 [file Table_2.docx]

**Supplemental Table S2.** Associations between selected sequenced miRNAs and biomarkers of bone metabolism. The table only includes miRNAs that were differentially expressed between samples with low and sufficient estrogen levels. Data are described as the estimated slope (β) with 95% CI, z values, and *p* values. The association of miRNAs with the concentrations of biomarkers is presented as β, where positive values indicate a positive association of the miRNA with the biomarker and vice versa. The statistical model was adjusted for FSH. miRNAs are sorted alphabetically.

| **miRNA** | **Biomarker** | **Estimate (**β**)** | **2.5% CI** | **97.5% CI** | **Z value** | **p-value** | **BH-adjusted p-value** |
| --- | --- | --- | --- | --- | --- | --- | --- |
| **let-7d-3p** | βCTX | 1,03 | -0,01 | 2,08 | 1,933 | 0,053 | 0,348 |
|  | Dkk1 | 0,01 | -0,01 | 0,03 | 1,021 | 0,307 | 0,706 |
|  | OPG | -0,07 | -0,21 | 0,06 | -1,1 | 0,271 | 0,641 |
|  | P1NP | 0,01 | 0 | 0,01 | 1,128 | 0,259 | 0,63 |
|  | Sclerostin | 0 | -0,01 | 0,01 | 0,121 | 0,904 | 0,96 |
| **miR-100-5p** | βCTX | 1,23 | -0,58 | 3,04 | 1,331 | 0,183 | 0,503 |
|  | Dkk1 | 0,02 | -0,01 | 0,05 | 1,587 | 0,112 | 0,484 |
|  | OPG | -0,04 | -0,28 | 0,2 | -0,341 | 0,733 | 0,904 |
|  | P1NP | 0,01 | 0 | 0,03 | 1,674 | 0,094 | 0,449 |
|  | Sclerostin | 0 | -0,02 | 0,01 | -0,353 | 0,724 | 0,904 |
| **miR-122-5p** | βCTX | 1,57 | -1,95 | 5,08 | 0,873 | 0,383 | 0,753 |
|  | Dkk1 | 0,04 | -0,02 | 0,1 | 1,397 | 0,162 | 0,502 |
|  | OPG | -0,12 | -0,59 | 0,35 | -0,502 | 0,616 | 0,895 |
|  | P1NP | 0,02 | -0,01 | 0,06 | 1,492 | 0,136 | 0,5 |
|  | Sclerostin | 0 | -0,05 | 0,05 | 0,003 | 0,998 | 0,998 |
| **miR-1278** | βCTX | 11,7 | 4,22 | 19,18 | 3,065 | 0,002 | 0,033 |
|  | Dkk1 | 0,18 | 0,06 | 0,29 | 3,046 | 0,002 | 0,033 |
|  | OPG | -0,45 | -1,1 | 0,2 | -1,352 | 0,176 | 0,502 |
|  | P1NP | 0,04 | 0,02 | 0,06 | 4,307 | 0 | 0,001 |
|  | Sclerostin | 0,06 | 0 | 0,12 | 2,104 | 0,035 | 0,25 |
| **miR-1304-5p** | βCTX | 5,05 | -0,77 | 10,87 | 1,7 | 0,089 | 0,449 |
|  | Dkk1 | -0,02 | -0,24 | 0,2 | -0,213 | 0,831 | 0,943 |
|  | OPG | -0,34 | -0,79 | 0,12 | -1,45 | 0,147 | 0,5 |
|  | P1NP | 0,04 | -0,02 | 0,09 | 1,379 | 0,168 | 0,502 |
|  | Sclerostin | -0,01 | -0,05 | 0,04 | -0,401 | 0,688 | 0,904 |
| **miR-132-5p** | βCTX | -1,91 | -4,04 | 0,22 | -1,759 | 0,079 | 0,449 |
|  | Dkk1 | 0 | -0,05 | 0,05 | -0,062 | 0,951 | 0,974 |
|  | OPG | -0,15 | -0,36 | 0,07 | -1,349 | 0,177 | 0,502 |
|  | P1NP | -0,01 | -0,02 | 0,01 | -0,991 | 0,322 | 0,72 |
|  | Sclerostin | 0 | -0,02 | 0,01 | -0,368 | 0,713 | 0,904 |
| **miR-195-5p** | βCTX | 0,22 | -1,21 | 1,65 | 0,306 | 0,759 | 0,904 |
|  | Dkk1 | 0 | -0,02 | 0,02 | 0,212 | 0,832 | 0,943 |
|  | OPG | -0,06 | -0,2 | 0,08 | -0,875 | 0,381 | 0,753 |
|  | P1NP | 0 | -0,02 | 0,01 | -0,896 | 0,37 | 0,753 |
|  | Sclerostin | 0 | -0,01 | 0,01 | 0,672 | 0,502 | 0,819 |
| **miR-196a-5p** | βCTX | 2,54 | -3,24 | 8,32 | 0,86 | 0,39 | 0,753 |
|  | Dkk1 | 0,07 | 0,03 | 0,12 | 3,327 | 0,001 | 0,025 |
|  | OPG | 0,25 | -0,69 | 1,18 | 0,518 | 0,605 | 0,895 |
|  | P1NP | 0,01 | -0,03 | 0,06 | 0,6 | 0,549 | 0,864 |
|  | Sclerostin | 0,02 | -0,03 | 0,06 | 0,703 | 0,482 | 0,819 |
| **miR-200a-3p** | βCTX | 1,78 | -1,95 | 5,5 | 0,936 | 0,35 | 0,747 |
|  | Dkk1 | 0,03 | -0,01 | 0,07 | 1,581 | 0,114 | 0,484 |
|  | OPG | -0,01 | -0,32 | 0,29 | -0,089 | 0,929 | 0,974 |
|  | P1NP | 0,01 | -0,02 | 0,03 | 0,433 | 0,665 | 0,904 |
|  | Sclerostin | 0 | -0,02 | 0,02 | 0,187 | 0,852 | 0,952 |
| **miR-24-1-5p** | βCTX | -6,71 | -11,53 | -1,88 | -2,722 | 0,006 | 0,079 |
|  | Dkk1 | -0,02 | -0,18 | 0,13 | -0,298 | 0,766 | 0,904 |
|  | OPG | -1,11 | -1,67 | -0,56 | -3,939 | 0 | 0,003 |
|  | P1NP | -0,05 | -0,09 | -0,01 | -2,333 | 0,02 | 0,167 |
|  | Sclerostin | -0,04 | -0,08 | 0 | -2,19 | 0,029 | 0,22 |
| **miR-3120-3p** | βCTX | 1,88 | -2,76 | 6,53 | 0,794 | 0,427 | 0,789 |
|  | Dkk1 | 0,02 | -0,06 | 0,09 | 0,414 | 0,679 | 0,904 |
|  | OPG | 0,24 | -0,37 | 0,85 | 0,771 | 0,441 | 0,797 |
|  | P1NP | 0,02 | -0,01 | 0,04 | 1,306 | 0,191 | 0,508 |
|  | Sclerostin | 0 | -0,03 | 0,04 | 0,158 | 0,874 | 0,953 |
| **miR-422a** | βCTX | 1,29 | 0,25 | 2,32 | 2,441 | 0,015 | 0,138 |
|  | Dkk1 | -0,01 | -0,02 | 0,01 | -0,673 | 0,501 | 0,819 |
|  | OPG | 0,05 | -0,09 | 0,2 | 0,715 | 0,475 | 0,819 |
|  | P1NP | 0,01 | 0 | 0,02 | 3,229 | 0,001 | 0,026 |
|  | Sclerostin | 0 | -0,01 | 0,01 | 0,428 | 0,669 | 0,904 |
| **miR-424-3p** | βCTX | 1,68 | -0,54 | 3,91 | 1,482 | 0,138 | 0,5 |
|  | Dkk1 | -0,01 | -0,04 | 0,03 | -0,324 | 0,746 | 0,904 |
|  | OPG | 0 | -0,23 | 0,23 | -0,035 | 0,972 | 0,984 |
|  | P1NP | 0,01 | 0 | 0,03 | 1,503 | 0,133 | 0,5 |
|  | Sclerostin | 0 | -0,02 | 0,01 | -0,53 | 0,596 | 0,895 |
| **miR-505-5p** | βCTX | 7,67 | -1,34 | 16,68 | 1,669 | 0,095 | 0,449 |
|  | Dkk1 | -0,01 | -0,19 | 0,17 | -0,14 | 0,889 | 0,956 |
|  | OPG | 0,11 | -0,57 | 0,79 | 0,311 | 0,756 | 0,904 |
|  | P1NP | 0,02 | -0,03 | 0,08 | 0,838 | 0,402 | 0,759 |
|  | Sclerostin | -0,06 | -0,14 | 0,02 | -1,382 | 0,167 | 0,502 |
| **miR-550a-3-5p** | βCTX | -6,19 | -35,07 | 22,69 | -0,42 | 0,674 | 0,904 |
|  | Dkk1 | -0,19 | -0,59 | 0,21 | -0,932 | 0,352 | 0,747 |
|  | OPG | -2,75 | -7,19 | 1,7 | -1,212 | 0,226 | 0,581 |
|  | P1NP | -0,03 | -0,23 | 0,16 | -0,352 | 0,725 | 0,904 |
|  | Sclerostin | 0,02 | -0,12 | 0,16 | 0,277 | 0,782 | 0,911 |
| **miR-566** | βCTX | 3,2 | -2,21 | 8,62 | 1,159 | 0,246 | 0,616 |
|  | Dkk1 | 0 | -0,12 | 0,13 | 0,075 | 0,94 | 0,974 |
|  | OPG | -0,07 | -0,91 | 0,78 | -0,16 | 0,873 | 0,953 |
|  | P1NP | 0,01 | -0,03 | 0,05 | 0,701 | 0,483 | 0,819 |
|  | Sclerostin | 0,02 | -0,04 | 0,08 | 0,564 | 0,573 | 0,886 |
| **miR-619-5p** | βCTX | -2,6 | -5,59 | 0,39 | -1,704 | 0,088 | 0,449 |
|  | Dkk1 | -0,09 | -0,16 | -0,03 | -2,677 | 0,007 | 0,079 |
|  | OPG | -0,1 | -0,38 | 0,19 | -0,658 | 0,511 | 0,819 |
|  | P1NP | -0,01 | -0,03 | 0 | -1,471 | 0,141 | 0,5 |
|  | Sclerostin | -0,01 | -0,04 | 0,02 | -0,494 | 0,621 | 0,895 |
