# Supplementary material for: Menopausal Transition: Prospective Study of Estrogen Status, Circulating MicroRNAs, and Biomarkers of Bone Metabolism
Source: Front Endocrinol (Lausanne). 2022 May 13;13:864299. doi: 10.3389/fendo.2022.864299 (PMC9137039; doi:10.3389/fendo.2022.864299)
Supplement: Supplementary file 3 [file Table_3.docx]

**Supplemental Table S3.** Associations between osteomiR® miRNAs and estrogen levels. The table includes miRNAs selected by the osteomiR® kit. Data are described as IRR with 95% CIs, t ratios, and *p* values. The difference in miRNA concentrations between samples with low/sufficient estrogen levels is presented as an IRR, where values > 1 indicate an increased concentration in samples with low estrogen levels and vice versa. The statistical model was adjusted for FSH. miRNAs are sorted in ascending order based on *p* values.

| **miRNA** | **Contrast** | **IRR** | **2.5% CI** | **97.5% CI** | **t-ratio** | **p-value** | **BH-adjusted p-value** |
| --- | --- | --- | --- | --- | --- | --- | --- |
| **let-7b-5p** | Low / Effective | 1,12 | 0,76 | 2 | 0,598 | 0,554 | 0,615 |
| **miR-320a** | Low / Effective | 1,38 | 0,93 | 2 | 1,63 | 0,111 | 0,447 |
| **miR-375** | Low / Effective | 102,64 | 0,76 | 13921 | 1,908 | 0,064 | 0,447 |
| **miR-188-5p** | Low / Effective | 4,88 | 0,47 | 51 | 1,37 | 0,179 | 0,447 |
| **miR-152-3p** | Low / Effective | 0,71 | 0,43 | 1 | -1,451 | 0,155 | 0,447 |
| **miR-582-5p** | Low / Effective | 1,38 | 0,5 | 4 | 0,633 | 0,53 | 0,615 |
| **miR-144-5p** | Low / Effective | 0,9 | 0,69 | 1 | -0,786 | 0,437 | 0,615 |
| **miR-141-3p** | Low / Effective | 0,51 | 0,11 | 2 | -0,893 | 0,377 | 0,615 |
| **miR-127-3p** | Low / Effective | 1,11 | 0,39 | 3 | 0,199 | 0,843 | 0,843 |
| **miR-17-5p** | Low / Effective | 0,94 | 0,79 | 1 | -0,748 | 0,459 | 0,615 |
