# Supplementary material for: Menopausal Transition: Prospective Study of Estrogen Status, Circulating MicroRNAs, and Biomarkers of Bone Metabolism
Source: Front Endocrinol (Lausanne). 2022 May 13;13:864299. doi: 10.3389/fendo.2022.864299 (PMC9137039; doi:10.3389/fendo.2022.864299)
Supplement: Supplementary file 4 [file Table_4.docx]

**Supplemental Table S4.** Associations between osteomiR® miRNAs and biomarkers of bone metabolism. The table includes miRNAs profiled by the osteomiR® kit. Data are described as the estimated slope (β) with 95% CIs, z values, and *p* values. The association of miRNAs with the concentration of biomarkers is presented as the slope, where positive values indicate a positive association of the miRNA with the biomarker and vice versa. The statistical model was adjusted for FSH. miRNAs are sorted alphabetically.

| **miRNA** | **Biomarker** | **Estimate (**β**)** | **2.5% CI** | **97.5% CI** | **z-value** | **p-value** | **BH-adjusted p-value** |
| --- | --- | --- | --- | --- | --- | --- | --- |
| **let-7b-5p** | βCTX | 0,45 | -0,12 | 1,01 | 1,546 | 0,122 | 0,857 |
|  | Dkk1 | 0,01 | -0,01 | 0,02 | 0,693 | 0,488 | 0,998 |
|  | OPG | -0,04 | -0,14 | 0,06 | -0,72 | 0,472 | 0,998 |
|  | P1NP | 0 | 0 | 0,01 | 0,603 | 0,547 | 0,998 |
|  | Sclerostin | 0 | -0,01 | 0,01 | 0,035 | 0,972 | 0,998 |
| **miR-127-3p** | βCTX | 0,52 | -1,27 | 2,32 | 0,574 | 0,566 | 0,998 |
|  | Dkk1 | 0 | -0,02 | 0,03 | 0,333 | 0,739 | 0,998 |
|  | OPG | 0,02 | -0,2 | 0,23 | 0,155 | 0,877 | 0,998 |
|  | P1NP | 0 | -0,01 | 0,01 | -0,01 | 0,992 | 0,998 |
|  | Sclerostin | 0,01 | -0,01 | 0,02 | 1,023 | 0,306 | 0,998 |
| **miR-141-3p** | βCTX | -0,88 | -2,83 | 1,08 | -0,881 | 0,378 | 0,998 |
|  | Dkk1 | 0 | -0,03 | 0,04 | 0,056 | 0,955 | 0,998 |
|  | OPG | 0,02 | -0,24 | 0,28 | 0,155 | 0,877 | 0,998 |
|  | P1NP | 0 | -0,02 | 0,01 | -0,604 | 0,546 | 0,998 |
|  | Sclerostin | 0 | -0,01 | 0,01 | -0,215 | 0,83 | 0,998 |
| **miR-144-5p** | βCTX | -0,27 | -0,64 | 0,1 | -1,429 | 0,153 | 0,857 |
|  | Dkk1 | 0 | 0 | 0,01 | 0,514 | 0,607 | 0,998 |
|  | OPG | -0,02 | -0,06 | 0,03 | -0,694 | 0,487 | 0,998 |
|  | P1NP | 0 | -0,01 | 0 | -1,424 | 0,154 | 0,857 |
|  | Sclerostin | 0 | 0 | 0 | 0,182 | 0,856 | 0,998 |
| **miR-152-3p** | βCTX | 0,13 | -0,6 | 0,86 | 0,359 | 0,719 | 0,998 |
|  | Dkk1 | -0,01 | -0,02 | 0,01 | -0,784 | 0,433 | 0,998 |
|  | OPG | -0,02 | -0,13 | 0,09 | -0,36 | 0,719 | 0,998 |
|  | P1NP | 0 | 0 | 0,01 | 0,344 | 0,731 | 0,998 |
|  | Sclerostin | 0 | -0,01 | 0,01 | -0,084 | 0,933 | 0,998 |
| **miR-17-5p** | βCTX | -0,24 | -0,49 | 0,01 | -1,908 | 0,056 | 0,857 |
|  | Dkk1 | 0 | 0 | 0,01 | 0,944 | 0,345 | 0,998 |
|  | OPG | -0,01 | -0,05 | 0,02 | -0,797 | 0,425 | 0,998 |
|  | P1NP | 0 | 0 | 0 | -1,185 | 0,236 | 0,969 |
|  | Sclerostin | 0 | 0 | 0 | -0,002 | 0,998 | 0,998 |
| **miR-188-5p** | βCTX | 2,87 | -0,6 | 6,34 | 1,619 | 0,105 | 0,857 |
|  | Dkk1 | 0,03 | -0,03 | 0,1 | 0,923 | 0,356 | 0,998 |
|  | OPG | -0,19 | -0,97 | 0,59 | -0,475 | 0,635 | 0,998 |
|  | P1NP | 0,02 | -0,01 | 0,05 | 1,165 | 0,244 | 0,969 |
|  | Sclerostin | -0,01 | -0,03 | 0,02 | -0,344 | 0,731 | 0,998 |
| **miR-320a** | βCTX | 0,54 | -0,08 | 1,16 | 1,717 | 0,086 | 0,857 |
|  | Dkk1 | 0,01 | 0 | 0,02 | 1,695 | 0,09 | 0,857 |
|  | OPG | -0,02 | -0,12 | 0,08 | -0,346 | 0,729 | 0,998 |
|  | P1NP | 0 | 0 | 0,01 | 1,188 | 0,235 | 0,969 |
|  | Sclerostin | 0 | -0,01 | 0,01 | 0,349 | 0,727 | 0,998 |
| **miR-375** | βCTX | 10,91 | 1,67 | 20,15 | 2,313 | 0,021 | 0,857 |
|  | Dkk1 | 0,04 | -0,1 | 0,18 | 0,574 | 0,566 | 0,998 |
|  | OPG | 0,11 | -0,94 | 1,15 | 0,2 | 0,842 | 0,998 |
|  | P1NP | 0,06 | 0 | 0,11 | 1,994 | 0,046 | 0,857 |
|  | Sclerostin | -0,05 | -0,15 | 0,04 | -1,146 | 0,252 | 0,969 |
| **miR-582-5p** | βCTX | 0,28 | -1,21 | 1,77 | 0,369 | 0,712 | 0,998 |
|  | Dkk1 | 0 | -0,03 | 0,03 | -0,009 | 0,993 | 0,998 |
|  | OPG | 0 | -0,19 | 0,18 | -0,049 | 0,961 | 0,998 |
|  | P1NP | 0 | -0,01 | 0,02 | 0,326 | 0,744 | 0,998 |
|  | Sclerostin | 0 | -0,01 | 0,01 | 0,154 | 0,878 | 0,998 |
